# Long-term outcome after closure of an atrial shunt in patients aged 60 years or older with ischemic stroke: A nationwide, registry-based, case-control study^☆^

**DOI:** 10.1016/j.ijcchd.2022.100438

**Published:** 2023-01-05

**Authors:** Alexia Karagianni, Zacharias Mandalenakis, Savvas Papadopoulos, Mikael Dellborg, Peter Eriksson

**Affiliations:** a Center for Adults with Congenital Heart Disease (ACHD), Sahlgrenska University Hospital/Östra, Sweden; Institute of Medicine, Department of Molecular and Clinical Medicine/Cardiology, Sahlgrenska Academy, Gothenburg University, Sweden; b Department of Business Administration, School of Business, Economics and Law, University of Gothenburg, Sweden

**Keywords:** Atrial shunt, Cryptogenic stroke, Advanced age, Transcatheter intervention, Cerebrovascular event

## Abstract

**Background:**

According to the current guidelines, evidence of the effects of transcatheter closure in patients aged ≥60 years with an atrial shunt and cryptogenic stroke is still limited.

**Methods:**

Using Swedish health registries, patients aged ≥60 years who had previously developed a cryptogenic cerebrovascular event and undergone transcatheter closure were identified. Patients with atrial fibrillation were excluded, and the remaining patients were propensity score-matched with patients of the same age and risk profile who had only undergone medical treatment and with controls from the general population. They were then followed up until 2017 (mean period of 7.1 ± 3.9 years).

**Results:**

In total, 100 patients of the intervention group were matched with 100 patients of the medical treatment group and with 100 controls and followed up. The hazard ratio for a recurrent ischemic stroke in the intervention group compared with the medical treatment group was 0.8 (95% confidence interval, 0.3–2.1), and that compared with the controls was 2.3 (95% confidence interval, 0.6–8.9). Atrial fibrillation occurred at the same rate in the two treatment groups (odds ratio, 0.8; 95% confidence interval, 0.4–1.7). However, patients in the intervention group developed vascular disease at a lower rate (odds ratio, 0.5; 95% confidence interval, 0.25–0.85).

**Conclusions:**

Patients aged ≥60 years with cryptogenic stroke may undergo transcatheter closure of an atrial shunt after thorough screening for other potential causes of stroke. The incidence of vascular disease seems to be mitigated in these patients relative to medically treated patients.

## Highlights

• Transcatheter closure of an atrial shunt in advanced-aged patients is applicable.
• The risk of recurrent stroke in patients with shunt is higher compared to controls.
• The risk of new-onset atrial fibrillation is increased in patients ≧60after closure.
• Advanced-aged patients develop less vascular disease after closure of the shunt.

## Abbreviations

*PFO*: patent foramen ovale
*ASD*: atrial septal defect
*CVE*: cerebrovascular event
*NPR*: National Patient Register
*SPDR*: Swedish Prescribed Drug Register
*ICD*: International Classification of Diseases
*TIA*: transient ischemic attack

## 1 Introduction

Three randomized studies and the extension of previous randomized studies published in recent years have shown the benefit of patent foramen ovale (PFO) closure in selected patients compared with medical treatment to avoid a recurrent ischemic stroke [[1], [2], [3], [4], [5]], in contrast to the negative findings of previous randomized studies [6,7]. Only younger patients aged <60 years with a PFO and an otherwise unexplained ischemic stroke were included in those positive trials, and a clear benefit of PFO closure over medical treatment alone was found. Therefore, the effect of PFO or small atrial septal defect (ASD) closure in advanced-age patients (≥60 years of age) is not known.

The underlying cause of stroke changes with age because of the development of comorbidities and progression of vascular pathology; thus, the risk–benefit equation of atrial shunt closure might not be the same as randomized studies have shown in younger patients [[1], [2], [3], [4]]. Moreover, diagnosis of an atrial shunt-induced cryptogenic stroke in older patients may require further and more intensive investigation than in younger patients [[8], [9], [10]] We therefore used several Swedish registries to investigate and compare the risk of recurrent ischemic stroke in patients aged 60 years or older with a previous ischemic cerebrovascular event (CVE) who underwent transcatheter closure of an atrial shunt with that in medically treated patients and subsequently with the incidence of an index ischemic stroke in matched controls without ischemic CVE and a known atrial shunt. We also studied the incidence of atrial fibrillation, cardiovascular comorbidities, and bleeding events in the intervention, medical treatment, and population control groups.

## 2 Methods

### 2.1 Data source

This retrospective registry-based nationwide cohort study was designed using the National Patient Register (NPR), the Cause of Death Register, and the Swedish Prescribed Drug Register (SPDR). The NPR contains complete data since 1987, and hospital outpatient clinics have also reported to the register since 2001.

The Cause of Death Register contains information regarding the date and causes of death of all individuals who died in Sweden. The SPDR contains information on all prescribed drugs purchased at pharmacies in Sweden since 2005.

### 2.2 Study population

The NPR was searched to identify all individuals diagnosed with an atrial shunt from January 1, 1997 to December 31, 2016. Atrial shunts were defined as ASD and PFO, considering that both have the same International Classification of Diseases (ICD) diagnosis. We included patients with an isolated atrial shunt (ASD/PFO) who had developed an ischemic CVE, namely ischemic stroke or transient ischemic attack (TIA), before or on the same date as diagnosis of the atrial shunt.

Patients with an ischemic CVE and atrial shunt who underwent closure of the atrial shunt with a percutaneous transcatheter technique were identified, as were patients with an ischemic CVE and an atrial shunt who underwent medical treatment.

From this cohort, patients aged ≥60 years were included for further analysis. The population was divided into two groups: those who underwent the intervention treatment after diagnosis of the ischemic CVE and atrial shunt and those who received the standard medical treatment based on the decision determined at the multidisciplinary conference [11].

To avoid including patients with a large ASD, all patients from the intervention group with known atrial fibrillation at baseline were excluded.

Because the intervention and medical treatment groups exhibited substantial diversity, the intervention treatment group was matched with the medical treatment group (matched medical group) using propensity scores without replacement and with adjustment for age, sex, and cardiovascular comorbidities (diabetes mellitus, hypertension, atrial fibrillation, and cardiovascular disease).

Additionally, each patient from the intervention group was matched 1:1 with a control individual of the same sex and year of birth and similar cardiovascular comorbidities from the Total Population Register (control group). Control individuals with a previous ischemic CVE and/or diagnosis of congenital heart disease were excluded. The control individuals were matched with the patients according to the date of the transcatheter intervention.

The follow-up duration was 1–20 years (until December 31, 2017). During this time, we identified those individuals who developed a recurrent/index ischemic stroke, new-onset atrial fibrillation, bleeding events, and cardiovascular comorbidities. In addition, we identified in the SPDR all antithrombotic medications that were purchased at the end of the study. Diagnosis of TIA during follow-up was not included as an outcome because of the subjectiveness of TIA diagnosis.

Patients who died after the diagnosis of ischemic stroke and atrial shunt, along with individuals who died in the control group, were identified, and the cause of death was recruited from the Cause of Death Register.

### 2.3 Definitions

We defined patients with an isolated atrial shunt as those with at least one hospital discharge and outpatient visits or a death certificate with a registered ICD diagnosis of Q211 (ICD-10, since 1997) or 746.42 or 745F (ICD-8 and ICD-9, respectively).

The operation code included in the study was FFC22. Patients with operation code FFC22 along with other operation codes related to congenital heart disease were excluded from the study.

The ICD diagnoses of ischemic stroke, TIA, atrial fibrillation, hypertension, diabetes mellitus, and cardiovascular disease (including coronary artery disease, myocardial infarction, and heart failure) according to ICD-8, ICD-9, and ICD-10 codes are presented in Supplementary Table 1.

The codes for antiplatelet and anticoagulant agents obtained from the SPDR are summarized in Supplementary Table 2.

The study baseline was defined as the date on which patients in the standard medical treatment group were diagnosed with an atrial shunt and the date on which patients in the intervention and control groups underwent the transcatheter intervention treatment. Therefore, the baseline characteristics refer to these dates.

The antithrombotic agents were categorized into antiplatelets and anticoagulants.

The primary endpoint of the study was recurrent ischemic stroke, defined as the second hospitalization for ischemic stroke or death due to ischemic stroke in the NPR or Cause of Death Register, occurring at least 4 weeks after the index ischemic stroke and atrial shunt diagnosis.

The secondary endpoints were new-onset atrial fibrillation (defined as an atrial fibrillation diagnosis in the NPR, after diagnosis of the index CVE and atrial shunt, or ≥48 h after closure of the atrial shunt), cardiovascular comorbidities, and major bleeding (including gastrointestinal bleeding and cerebral hemorrhage occurring after the baseline for each group).

### 2.4 Statistics

All data analyses were performed using Stata (StataCorp, College Station, TX, USA). To check the similarity of the intervention group and medical group, the mean difference *t*-test was performed. The test showed that the two treatment groups had significant differences in age, diabetes, hypertension, coronary heart disease, myocardial infarction, heart failure, and atrial fibrillation. We therefore performed propensity score matching of the two treatment groups. The propensity score was established using logistic regression with 1:1 matching, where each patient from the intervention treatment group was matched to a patient from the standard medical treatment group, within common support, without replacement, and with a caliper distance of 0.2. The propensity score model was adjusted for age, sex, coronary heart disease, myocardial infarction, heart failure for the treatment groups, diabetes, atrial fibrillation, and hypertension. Continuous variables are expressed as mean ± standard deviation, and categorical variables are expressed as count and percentage. Odds ratios (ORs) and 95% confidence intervals were estimated at follow-up. A Cox regression model using univariate analysis was used to estimate the hazard ratios and 95% confidence intervals. We performed a stratified log rank test for the intervention versus medical group and for the intervention versus control group to examine whether the groups were equal in terms of events for recurrent/index ischemic stroke. Because of the high value of the log rank test for the intervention versus medical group, we performed Cox regression including time-dependent variables. The cumulative incidence of ischemic stroke is presented for the patients and their controls.

### 2.5 Ethics

This study was approved by the Gothenburg Regional Research Ethics Board and was performed in accordance with the Declaration of Helsinki (registration number Gpg 912–16, T616-18). Patient consent for publication was not required.

## 3 Results

**Fig. 1:**
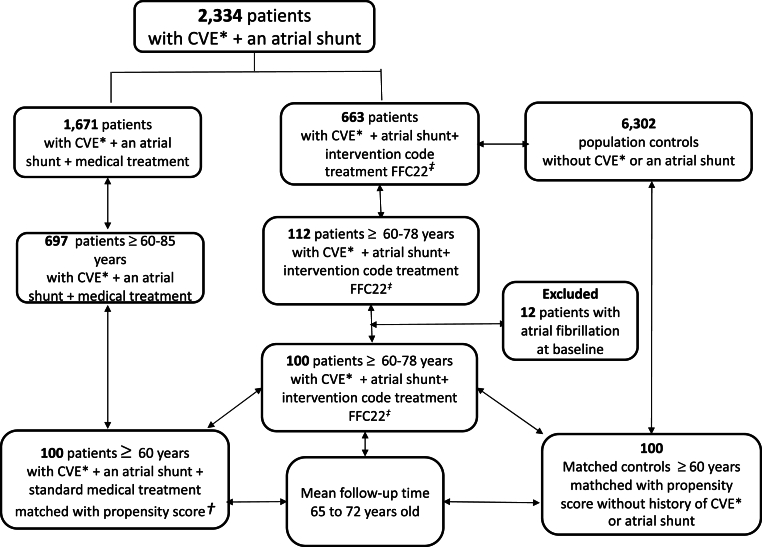
Flow chart of the study population. *CVE: Ischemic cerebrovascular event: **Propensity score adjusted for age, gender and cardiovascular risk factors: *** FFC22: Transcatheter closure of an atrial septal defect.

In total, 809 patients aged ≥60 years with a prior ischemic CVE and diagnosis of an atrial shunt from January 1997 to December 2016 were identified from the NPR. The intervention group comprised 112 patients, and the medical group comprised 697 patients (Fig. 1). Twelve patients from the intervention group were excluded because of known atrial fibrillation at baseline. The 100 remaining patients were matched by propensity scores with 100 patients from the medical group and 100 population controls.

The baseline characteristics of the two treatment groups (intervention and medical groups) before and after propensity score matching and the baseline characteristics of the population control group are summarized in Table 1. The follow-up period for patients and controls extended to December 2017 (mean period of 7.1 ± 3.9 years). The mean age of the study population was 65 ± 4.6 years at baseline and 72 ± 5 years at the end of the study.

**Table 1:** Baseline Characteristics for individuals ≧60 years.

	All patients (n = 809 a)		Propensity score matched population (n = 300^b^)		
	Medical group (n = 697)	Intervention group (n = 112)	Intervention group (n = 100)	Medical group (n = 100^c^)	Control group (n = 100^d^)
Age mean (SD,min-max)	68.7 (6, 60–85)	65.4 (4.6, 60–78)	65 (4.1, 60–78)	65.1 (4.6, 60–81)	64.9 (4.3, 60–78)
Men n (%)	388 (56)	71 (63)	64 (64)	63 (63)	65 (65)
Diabetes n (%)	73 (10.5)	4 (3.6)	2 (2)	1 (1)	2 (2)
Hypertension n (%)	384 (55)	39 (35)	31 (31)	31 (31)	31 (31)
Coronary disease n (%)	138 (20)	10 (8.9)	8 (8)	10 (10)	8 (8)
Myocardial infarction n (%)	56 (8)	1 (0.9)	0	0	0
Heart failure n (%)	86 (12)	3 (2.7)	0	0	1
Atrial fibrilaltion n (%)	145 (21)	12 (10.7)	–		
Cerebral hemorrhage n (%)	12 (1.7)	1 (0.9)	0	0	0
Gastrointestinal beeding n (%)	11 (1.6)	0 (0)	0	1 (1)	1 (1)
Ischemic stroke n (%)	473 (68)	81 (72)	73 (73)	55 (55)	–
TIA n (%)	224 (32)	31 (28)	27 (27)	35 (35)	–
MultipleCVE:s	57 (8.1)	16 (14)	15 (15)	10 (10)	–
Age at the end of the study mean (SD,min-max)	75 (6.1, 60–87	72.4 (5, 61–87)	72.3 (4.8, 61–87)	73 (5.8, 61–87)	71.8 (5.4, 63–87)

a *The intervention and medical group before excluding patients with atrial fibrillation at baseline*.

b *Population propensity score matched after excluded atrial fibrillation at baseline*.

c Propensity score included age, gender and comorbidities (diabetes, hypertension, coronary artery disease, heart failure, myocardial infarction and atrial fibrillation).

d Propensity score included age, gender and comorbidities (diabetes, hypertension, coronary artery disease, myocardial infarction and atrial fibrillation).

Nine patients in the intervention group died during follow-up, and two of them had developed a recurrent ischemic stroke and were included in the analysis of the primary endpoint. Fifteen patients in the matched medical group died during follow-up, and three of them had developed a recurrent ischemic stroke. Thirteen patients in the control group died during follow-up, but only one had developed an index ischemic stroke.

### 3.1 Recurrent ischemic stroke

**Fig. 2:**
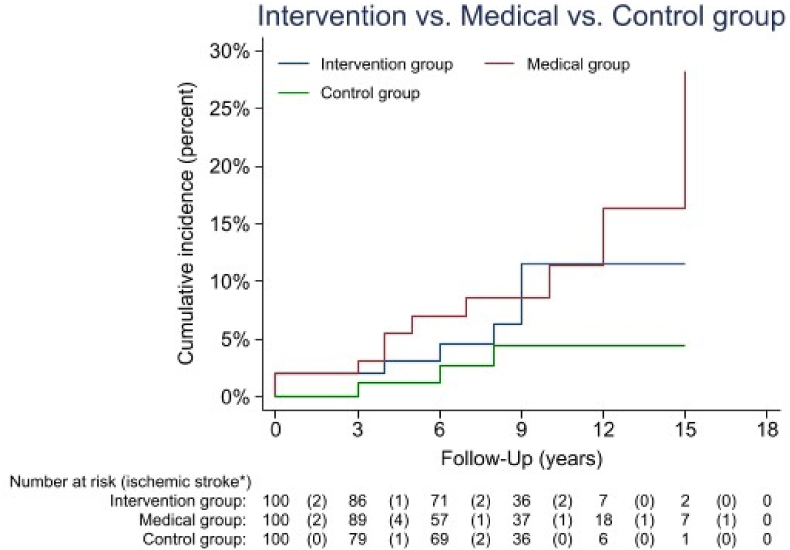
Recurrent/index stroke on follow-up for the intervention, and propensity score matched medical and population controls group. Intervention/medical group: Log rank test p-value: 0,5 **, hazard ratio -0.3; 95% CI, -1.2- 0.7), Intervention/control group: Log rank test p-value: 0.2, hazard ratio 2.3; 95% CI, 0.6–8.9 *Ischemic stroke: recurrent ischemic stroke for intervention and medical groups and index ischemic stroke for control group at follow-up**Because of the high value of the stratified log rank test for intervention vs medical group, we performed Cox regression including time dependent variable.

Seven (7%) patients in the intervention group developed a recurrent ischemic stroke, excluding patients with atrial fibrillation at baseline. In total, 70 (10%) of 697 patients in the medical group developed a recurrent ischemic stroke, and 10 (10%) of 100 patients in the matched medical group were found to have developed a recurrent ischemic stroke. In the control group, three (3%) individuals developed an index ischemic stroke (Table 2). The risk of recurrent ischemic stroke was 1 per 100 patient-years in the intervention group versus 1.4 in the matched medical group, and the risk of an index ischemic stroke in the control group was 0.4 per 100 patient-years. The univariate analysis showed that the hazard ratio of a recurrent ischemic stroke in the intervention group compared with the medical group was 0.8 (95% confidence interval, 0.3–2.1), and that after performing Cox regression including time-dependent variables was −0.3 (95% confidence interval, −1.2 to 0.7). The risk of a recurrent ischemic stroke in the intervention group compared with the risk of an index ischemic stroke in the control group was higher (hazard ratio, 2.3; 95% confidence interval, 0.6–8.9) (Fig. 2). The mean age of patients with a recurrent ischemic stroke in the intervention group was 73 ± 5.5 years, that in the matched medical group was 71 ± 6.5 years, and that in the control group was 73 ± 4 years.

**Table 2:** Follow-up: Long-term outcomes of population.

All patients ≧ 60 years (n = 809^a^)			Propensity Score Matched population ≧60 years (n = 300^b^)			
New-onset^e^	Intervention/Medical group	0dds ratio (95%)	Intervention/Medical group^c^	0dds ratio (95%)	Intervention/Control group^d^	Odds ratio (95%)
Diabetes n (%)	8 (7.1)/40 (5.7)	1.3 (0.5–2.8)	8 (8)/7 (7)	1.2 (0.4–3.3)	8 (8)/6 (6)	1.4 (0.4–4)
Hypertension n (%)	10 (9)/96 (14)	0.6 (0.3–1.2)	10 (10)/16 (16)	0.6 (0.3–1.4)	10 (10)/18(18)	0.5 (0.2–1.2)
Coronary disease n (%)	10 (9)/62 (9)	1 (0.4–2)	8 (8)/8 (8)	1 (0.4–2.3)	8 (8)/6(6)	1.4 (0.4–4)
Myocardial infarction n (%)	5 (4.5)/32 (4.6)	1 (0.3–2.6)	4 (4)/5 (5)	0.8 (0.2–3)	5 (5)/2 (2)	2.3 (0.5–13.6)
Heart failure n (%)	7 (6.3)/68 (9.8)	0.6 (0.2–1.4)	5 (5)/13 (13)	0.4 (0.1–1)	5 (5)/2 (2)	2.3 (0.5–13.6)
Atrial fibrillation^f^ n (%)	16 (14)/109 (15.6)	0.9 (0.5–1.6)	16 (16)/19 (19)	0.8 (0.4–1.7)	16 (16)/10 (10)	1.7 (0.7–4)
-AF in 60 days post op			4 (3.5)			
-AF after 60 days post op			12 (10.7)			
Cerebral hemorrhage n (%)	0 (0)/17 (2.4)	–	0 (0)/2 (2)	–	0 (0)/5 (5)	–
Gastrointestinal bleeding n (%)	2 (1.8)/21 (3)	0.6 (0.07–2.5)	2 (2)/3 (3)	0.7 (0.1–4)	2 (2)/4 (4)	0.5 (0.09–2.7)
Ischemic stroke n (%)	8 (7.1)/70 (10)	0.7 (0.3–1.5)	7 (7)/10 (10)	0.7 (0.2–1.9)	7 (7)/3 (3)	2.4 (0.6–9.7)

a The intervention and medical group before excluding patients with atrial fibrillation at baseline.

b Population propensity score matched after excluded atrial fibrillation at baseline.

c Propensity score included age, gender and comorbidities (diabetes, hypertension, coronary artery disease, heart failure, myocardial infarction and atrial fibrillation).

d Propensity score included age, gender and comorbidities (diabetes, hypertension, coronary artery disease, myocardial infarction and atrial fibrillation).

e *No previous reported diagnosis in the NPR or the Cause of Death Register*.

f *New-onset atrial fibrillation diagnosed after the baseline and during follow-up time*.

### 3.2 New-onset atrial fibrillation

Atrial fibrillation was identified in 16 (16%) patients in the intervention group, excluding patients with atrial fibrillation at baseline (Table 2). Atrial fibrillation was diagnosed in 4 patients during the first 2 months after the intervention and in 12 patients at least 2 months after the intervention. New-onset atrial fibrillation occurred in 109 (16%) patients in the medical group and 19 (19%) in the matched medical group (Table 2). Ten (10%) control individuals developed atrial fibrillation during the follow-up (Table 2). In total, 2 of 7 patients with recurrent ischemic stroke developed atrial fibrillation in the intervention group, 3 of 10 patients developed atrial fibrillation in the matched medical group, and 1 of 3 patients in the control group with an index ischemic stroke was diagnosed with atrial fibrillation during follow-up.

### 3.3 Cardiovascular comorbidities

New-onset hypertension developed at a lower level in the intervention group than in both the total medical group and matched medical group (OR, 0.6; 95% confidence interval, 0.3–1.2 and OR, 0.6; 95% confidence interval, 0.3–1.4). During follow-up, hypertension was diagnosed in more patients in the control group than intervention group (OR, 0.5; 95% confidence interval, 0.2–1.2). The incidence of diabetes, coronary disease, and myocardial infarction was similar among the intervention, matched medical treatment, and control groups (Table 2).

However, the incidence of vascular disease during follow-up, including hypertension, myocardial infarction, coronary artery disease, heart failure, and cerebrovascular disease (ischemic stroke and cerebral hemorrhage), was significantly higher in the matched medical group than intervention group (42/100 vs. 61/100; OR, 0.5; 95% confidence interval, 0.25–0.85).

### 3.4 Antithrombotic agents

More antiplatelet agents were prescribed and purchased in the intervention group than medical group, whereas more anticoagulant agents were purchased in the medical group (Supplementary Table 3).

#### 3.5.1 Major bleeding

Bleeding, including gastrointestinal bleeding and cerebral hemorrhage, occurred at a lower rate in the intervention group than total medical group and matched medical group (OR for intervention/medical group, 0.3; 95% confidence interval, 0.1–1.3 and OR for intervention/matched medical group, 0.4; 95% confidence interval, 0.07–2). The control group developed major bleeding at a higher rate than the intervention group (OR, 0.2; 95% confidence interval, 0.04–0.98) (Table 2).

## 4 Discussion

The present study included patients aged ≥60 years who underwent transcatheter closure of an atrial shunt before publication of the above-mentioned randomized studies and the current guidelines, and the patients were followed up for a mean of 7 years. Using Swedish registries, we found that the relative risk of recurrent ischemic stroke tended to be lower in the intervention group than in the medical group even after excluding patients with known atrial fibrillation at baseline. However, the relative risk of recurrent stroke in the intervention group was higher than the risk of an index stroke in the population control group. This suggests that prior ischemic stroke is also a major risk factor for a recurrent ischemic stroke in patients who have been treated by transcatheter closure of an atrial shunt. In studies that included patients with not only cryptogenic stroke, the presence of a prior ischemic stroke was by itself a risk factor for a recurrent stroke [12,13]. Although one may assume that this does not apply to patients with a cryptogenic stroke and an atrial shunt that has been closed, the current data do not support this assumption.

Previous randomized studies of device-closure of atrial shunts after a cryptogenic stroke included only patients aged up to 60 years, and the maximum follow-up time was 5 years. The risk of recurrent ischemic stroke in these studies was 0.4–0.6 per 100 patient-years [3,5]. Current guidelines do not recommend intervention treatment for closure of an atrial shunt after a cryptogenic CVE in patients aged >60 years [14].

The absolute risk of a recurrent ischemic stroke was higher in both the intervention group and medical treatment group than in randomized studies (7% vs. up to 3.6%) [[1], [2], [3], [4]]. However, the prevalence of stroke among adults aged ≥60 years is around 6% in those aged 60–79 years and 12% in those aged >80 years [15]. Chronological aging is the single most important risk factor for stroke [16]. However, using cut-offs of chronological age may introduce difficulties into everyday clinical practice. During the last few decades, studies have shown that aging depends on biological predictors rather than chronological time [17].

Observational studies have shown the effectiveness of PFO closure in older patients in terms of a low rate of periprocedural complications similar to the low rate in younger patients. However, the incidence of recurrent ischemic events was higher than that in younger patients [18,19].

In the present study, the risk of new-onset atrial fibrillation was similar in the two treatment groups (intervention and medical group) but higher than that in the control group. In 75% of patients, atrial fibrillation presented more than 2 months after the intervention. In contrast, previous studies that included only younger patients showed a higher incidence of atrial fibrillation shortly after the intervention (up to 45 days) than in medically treated patients [10,[20], [21], [22]]. Randomized studies showed a lower incidence of atrial fibrillation in the intervention group (4.6%–6.8%) than in the present study [1,2]. However, the prevalence of atrial fibrillation increases with age (1.9% by 60 years, 4.6% by 70 years, and 12.5% by 80 years) [23]. Therefore, considering that patients and controls were at least 60 years old at baseline in the present study, the high incidence rate of atrial fibrillation during the mean follow-up of 7 (1–20) years can be partially explained by the aging of the population. Furthermore, occult atrial fibrillation that can cause cryptogenic stroke may have been underestimated in the present study. Notably, 12.5% of patients with new-onset atrial fibrillation in the intervention group developed recurrent ischemic stroke. This suggests that occult atrial fibrillation is a significant but not the only cause of recurrent ischemic stroke in older patients who have undergone closure of an atrial shunt. Thus, longer Holter electrocardiographic analyses should be recommended in older patients with an ischemic CVE of unknown source. Moreover, observational studies have shown that residual shunting after transcatheter closure of an atrial shunt may be the major cause of recurrent ischemic stroke [24,25]. In the present study, the incidence of residual shunting after closure of the atrial shunt could not be identified from the registries.

The intervention group had lower rate of comorbidities than the medical group at baseline. This suggests that patients from the intervention group were well selected and otherwise quite healthy or adequately treated.

It is also worth noting that patients in the intervention group developed vascular disease at a significantly lower rate than the matched medical group during follow-up. We consider this to be a post-hoc tertiary endpoint, and it could be claimed that this was due to the presence of the atrial shunt in the medical group and/or the prolonged medical treatment. Thus, a presumed benefit of the intervention treatment is avoidance of thromboembolic cardiovascular disease in patients aged ≥60 years with a cryptogenic CVE and an atrial shunt.

### 4.1 Major bleeding and antithrombotic agents

The absolute risk of major bleeding, including gastrointestinal and cerebrovascular bleeding, was low in both treatment groups and similar to the events in the REDUCE randomized study [1]. The higher rate of antithrombotic agents, especially anticoagulants, purchased by the patients in the medical group can at least partially explain the lower incidence of bleeding events in the intervention than medical group. At present, the choice of an anticoagulant agent over an antiplatelet agent in patients who have developed a cryptogenic CVE because of an atrial shunt has not been well studied in patients of advanced age, although anticoagulant agents are recommended over antiplatelet agents in younger patients [26] when applicable in patients who have developed a cryptogenic CVE because of an atrial shunt.

## 5 Limitations

Our findings should be interpreted in the context of the limitations of this study. First, this was a cohort study using national registries; therefore, the risk of diagnostic misclassification exists. However, the diagnoses of ischemic stroke and atrial shunt have been shown to have high validity (85%–95%) [27], and the same applies to the SPDR [28]. Moreover, the validity of a myocardial infarction diagnosis in patients with congenital heart disease, including atrial shunts, is as high as 88% [29].

The number of individuals and events in each group was small and does not give enough power to the results of this study. Nevertheless, the older age category of the present study was not included in previous randomized studies and is a novel aspect of our research.

Furthermore, although ASD and PFO are two different conditions, they have the same ICD code. However, by including only patients who developed an ischemic CVE and were then diagnosed with an atrial shunt, and by excluding patients with known atrial fibrillation at baseline, the numbers of patients with a large ASD and remodeled atria are limited.

Finally, the SPDR was introduced in July 2005; therefore, patients who purchased antithrombotic agents before the introduction of the registry were not included.

## 6 Conclusions

In this nationwide cohort study, we found that patients aged ≥60 years with cryptogenic stroke may also undergo transcatheter closure of an atrial shunt, although the higher risk of recurrent stroke compared with population controls remains a concern. Thorough screening for other potential causes of stroke, especially occult atrial fibrillation, is essential. Intervention treatment is beneficial in mitigating the incidence of vascular disease relative to medical treatment. Further studies are essential to identify which patients aged ≥60 years are suitable for intervention treatment of an atrial shunt after an ischemic CVE and to investigate the low incidence of vascular disease in the aforementioned patients after intervention.

## Funding source

This work was supported by the Swedish state under an agreement between the Swedish government and county councils, the 10.13039/100001424ALF agreement (Grant Number: 236,611), and the 10.13039/501100003793Swedish Heart-Lung Foundation (Grant Numbers: 20,090,724 and 20180644).

## Declaration of competing interest

The authors declare the following financial interests/personal relationships which may be considered as potential competing interests:Mikael Dellborg reports financial support was provided by Swedish Heart and 10.13039/100002590Lung Association (https://www.hjart-lungfonden.se/om-oss/in-english/). Zacharias Mandalenakis reports financial support was provided by 10.13039/100001424ALF agreement.
