# Supplementary material for: Long-term outcome after closure of an atrial shunt in patients aged 60 years or older with ischemic stroke: A nationwide, registry-based, case-control study
Source: Int J Cardiol Congenit Heart Dis. 2023 Jan 5;11:100438. doi: 10.1016/j.ijcchd.2022.100438 (PMC11657852; doi:10.1016/j.ijcchd.2022.100438)
Supplement: Multimedia component 1 [file mmc1.docx]

**Supplementary material**

| **Supplementary Table 1: ICD diagnostic codes for the outcomes and co-morbidities** | | |  |  |
| --- | --- | --- | --- | --- |
|  |  |  | |  |
| **Diagnosis** | **ICD-8** | **ICD-9** | | **ICD-10** |
|  |  |  | |  |
| Stroke | 434 | 436 | | I63, I64 |
| TIA | 435 | 435 | | G45 |
|  |  |  | |  |
| Atrial Fibrillation | 427.92 | 427D | | I48 |
|  |  |  | |  |
| Ischemic Heart disease | 410,411,412,413,414 | 410,411,412,413,414 | | I20,I21,I22,I23,I24,I25 |
|  |  |  | |  |
| Myocardial infarction | 410 | 410 | | I21 |
|  |  |  | |  |
| Heart failure | 427.00 | 428 | | I50 |
|  |  |  | |  |
| Diabetes mellitus | 250 | 250 | | E10,E11, E12,E13,E14 |
|  |  |  | |  |
| Hypertension | 400,401,402,403,404 | 401,402,403,404,405 | | I10,I11,I12,I13,I14,I15 |
| Major bleeding* |  |  | | I61, K92.0,K92.1, K92.2 |
|  |  |  | |  |

**Major bleeding including gastrointestinal bleeding and cerebral hemorrhage*

| **Supplementary Table 2: ATC codes for antithrombotic agents** | | | |  |
| --- | --- | --- | --- | --- |
|  |  |  |  | |
| Anti-platelet agents | | Acetylsalicylsyra | B01AC06 | |
|  |  |  | B01AC30 | |
|  |  |  |  | |
|  |  | Clopidogrel | B01AC04 | |
|  |  |  |  | |
|  |  | Dipyridamol | B01AC07 | |
|  |  |  |  | |
| Anti-coagulant agents | | Warfarin | B01AA03 | |
|  |  |  |  | |
|  |  | Apixaban | B01AF02 | |
|  |  |  |  | |
|  |  | Rivaroxaban | B01AF01 | |
|  |  |  |  | |
|  |  | Dabigatran | B01AE07 | |

| **Supplementary Table 3** | | |  |  |  |  |  |  |  |  |
| --- | --- | --- | --- | --- | --- | --- | --- | --- | --- | --- |
|  | |  | |  |  |  |  |  |  |  |
|  |  | Individuals aged ≧ 60 years | | | | | | | | |
| Antithrombotic agents at  follow-up n (%) | | All patients (n=809) | | | | Propensity score matched population | | | | |
|  |  | Medical group (n=686∞) | | Intervention group (n=112) | | Intervention group (n=100) | | | Medical group (n=100) | Control group (n=100) |
| Antiplatelet agents * |  | 328(47) |  | 84 (75) |  | 81 (81) |  |  | 52 (52) | 27 (27) |
| Anticoagulant agents ** |  | 322 (46) |  | 27 (24.1) |  | 17 (17) |  |  | 38 (38) | 13 (13) |
| Antiplatelet and anticoagulant agents | | 14 (2) |  | 0 (0) |  | 0 |  |  | 3 (3) | 0 |
| No antithrombotic agents |  | 22 (3.2) |  | 1 (0.9) |  | 1 (1) |  |  | 5 (5) | 59 (59) |

**Acetylsalicylic acid, clopidogrel, and dipyridamole*

*** Warfarin, rivaroxaban, apixaban, and dabigatran*

*∞ The antithrombotic agents of 11 patients from the medical group are not included (SPDR introduces on July 2005)*
